# A longitudinal study of fruit juice consumption during preschool years and subsequent diet quality and BMI

**DOI:** 10.1186/s40795-020-00347-6

**Published:** 2020-05-14

**Authors:** Li Wan, Phani Deepti Jakkilinki, Martha R. Singer, M. Loring Bradlee, Lynn L. Moore

**Affiliations:** 1 Department of Medicine, Preventive Medicine and Epidemiology, Boston University School of Medicine, Boston, MA 02118 USA; 2 Currently: Massachusetts General Hospital Cancer Center, 149 13th Street, Boston, MA 02129 USA; 3 Currently: Centre for Chronic Disease Control, Public Health Foundation of India, C-1/52, 2nd Fl, Safdarjung Development Area, New Delhi, 110016 India

**Keywords:** Fruit juice, Fruit intake, Diet quality, Children, Adolescence

## Abstract

**Background:**

The role of fruit juice in pediatric dietary guidelines continues to be controversial, particularly with respect to concerns about unhealthy dietary habits and the potential promotion of excessive weight gain. The objective of the current study was to determine the association between preschool fruit juice consumption and the following outcomes during childhood and adolescence: whole and total fruit intake, diet quality, likelihood of meeting current dietary recommendations, and BMI change.

**Methods:**

The data were previously collected from 100 children enrolled in the Framingham Children’s Study at 3–6 years of age and subsequently followed for 10 years. Dietary data were collected annually using multiple sets of 3-day diet records. Compliance with dietary recommendations was estimated based on *2015–2020 Dietary Guidelines for Americans* and diet quality was measured using the associated Healthy Eating Index (HEI). Mixed linear and logistic regression models were used for statistical analyses.

**Results:**

Preschool children (3–6 years) who drank ≥1.0 (vs. < 0.5) cup of 100% fruit juice/day consumed 0.9 cups/day more total fruit (*p* < 0.0001) and 0.5 cups/day more whole fruit (*p* < 0.0001) during adolescence (14–18 years). Total HEI scores during adolescence for those with the highest preschool juice intakes were almost 6 points higher than those with the lowest fruit juice intakes (*p* = 0.0044). Preschoolers consuming < 0.5 cups/day of fruit juice had sharply declining whole fruit intake throughout childhood compared with those preschoolers consuming ≥1.0 cups/day who had stable intakes of whole fruit throughout childhood. Those children who consumed ≥0.75 cups/day of fruit juice during preschool (vs. less) were 3.8 times as likely to meet *Dietary Guidelines* for whole fruit intake during adolescence (*p* < 0.05). Finally, in multivariable models, there was no association between fruit juice consumption and BMI change throughout childhood.

**Conclusion:**

These data suggest that preschool consumption of 100% fruit juice is associated with beneficial effects on whole fruit intake and diet quality without having any adverse effect on BMI during childhood and into middle adolescence.

## Background

Fruit is a widely recognized source of a significant number of beneficial nutrients including vitamins, minerals, phytochemicals, and dietary fiber that have been associated with lower risks of cardiovascular disease and obesity [1]. For children and adolescents aged 2–18 years, guidelines from United States Department of Agriculture (USDA) recommend daily fruit intakes of 1–2 cups depending on age, sex, and physical activity level [2]. It is also recommended that at least half of daily total fruit intake for both children and adults be derived from whole fruit [3]. Data from *National Health and Nutrition Examination Surveys (NHANES)* show that fruit juice consumption among preschoolers peaked in the early 2000s [4] and then subsequently declined while whole fruit consumption slightly increased [5]. However, most children (particularly after the preschool years) still fail to consume the recommended amount of total fruit per day. Cross-sectional analyses of NHANES data from 2005 to 2010 show that whole and total fruit consumption declines with increasing age. For example, 4–8-year olds ate significantly more whole and total fruit than 9–13-year olds, who in turn consumed more than 14–18-year olds, whose mean total fruit intakes were half of the recommended amount [6].

The role of 100% fruit juice in total fruit intake amongst children, especially younger children, continues to be controversial [7]. Longstanding concerns about juice intake include its lower fiber content (compared with whole fruit), caloric density, and potential promotion of dental caries. Current American Academy of Pediatrics (AAP) guidelines recommend avoiding fruit juice in infants less than 1 year of age and encourage consumption of whole fruit rather than fruit juice throughout childhood and adolescence [8]. After 1 year of age, the AAP concurs that fruit juice may comprise up to half of the recommended total daily fruit intake but also recommends that intakes among 1 to 3-year old children should be limited to 4 oz (oz) per day and among those 4 to 6 years of age to 4–6 oz. per day.

Some earlier investigators have suggested that to address the rising rates of obesity, fruit juice should be eliminated from federal nutrition programs [9]. The AAP concurs that fruit juice restriction may be an effective strategy for reducing of energy imbalance in young children [8]. However, there is little evidence to support a link between juice consumption and childhood obesity. One early study found that preschoolers who consumed > 12 oz of fruit juice per day were at increased risk for excess weight gain [10] while another study published the same year found no such association [11]. Several reviews of the evidence in children have concluded that there is no independent or systematic contribution of 100% fruit juice to clinically-significant weight gain or obesity risk in children [12–14]. On the other hand, fruit juice is known to have beneficial antioxidant capacity [15] and children who consume it have been shown to have higher intakes of important micronutrients such as vitamins C and B6, potassium, riboflavin, magnesium, iron, and folate compared with non-consumers [16].

Most studies that have examined the association between fruit juice consumption and diet quality in children have been cross-sectional [13]. Whether fruit juice consumption in early childhood affects overall fruit intake and diet quality in later childhood and adolescence remains largely unknown. In this study, we have used data from the Framingham Children’s Study (FCS) in which children were enrolled at 3–6 years of age and followed for approximately 10 years to investigate the relation between greater consumption of 100% fruit juice during preschool and subsequent intakes of whole and total fruit, the overall likelihood of meeting dietary guidelines, overall diet quality, and change in BMI throughout childhood.

## Methods

These analyses are based on previously collected data. The FCS was a longitudinal study that enrolled 106 children from two-parent families with a 3–6 year-old child in 1987. The families were third and fourth generation descendants of subjects in the original Framingham Heart Study [17]. Of the original 106 families, 100 provided dietary data for the children at baseline (preschool) and throughout follow-up (adolescence). Diet, physical activity and other lifestyle factors were evaluated annually by means of interviews, questionnaires, and clinical measurements over a period of 10 years [18–20].

### Dietary data

Dietary data were collected annually using multiple sets of 3-day diet records. During early years of the study (prior to age 10), parents completed the diaries for the children, with input from other caregivers inside and outside of the family. A study nutritionist instructed each family in the completion of the diet record including accurate estimation of portion size. Nearly 90% of subjects completed diet records for eight or more of the 11 years in the study. Dietary data were analyzed for nutrient intake using the Nutrition Data System for Research (NDSR) of the University of Minnesota [21]. Mean servings per day at each age in the 30 USDA food groups were calculated by linking NDSR food codes with Pyramid Serving data files of the Continuing Survey of Food Intake by Individuals [22]. For our calculation of fruit juice, only 100% fruit juices and 100% juice blends such as 100% cranberry juice blends (i.e., blended with other 100% juices) were included. Part-juice beverages and tomato juice were excluded from fruit juices. Intake of whole fruit (including cut fruit) and juices are expressed as USDA-defined cup-equivalents per day. The most common types of juice beverages in the preschool years were apple and orange juices.

### Outcome variables

Each child’s intake of whole and total fruit throughout childhood was examined to determine whether the child met the recommendations for fruit intake at each age. Based on *Dietary Guidelines for Americans* (DGA) recommendations, the following levels were considered to meet guidelines for total fruit intake: 1 cup for 2–3 year-olds, 1–1½ cups for 4–8 years, 1½ cups for 9–13 years old, and 1½ cups for 14–18 year-old girls and 2 cups for 14–18 year-old boys [3]. Diet quality was based on the 2015 Healthy Eating Index (HEI-2015) total score which is designed to measure conformance with the 2015 USDA DGAs. HEI-2015 is comprised of 13 component scores with a maximum total score of 100. Two fruit outcomes are included: whole fruit and total fruit intake. As an overall measure of diet quality, the HEI-2015 has been shown to be both reliable and valid [23].

Each child’s height and weight were recorded at each annual clinic exam. Weight (to the nearest 1/4 pound) was measured using a standard counterbalance scale, and height was measured (to the nearest 1/4 in) using a measuring bar on the same scale. Body mass index (BMI) was calculated as weight in kilograms divided by height in meters squared.

### Statistical analysis

Children were categorized into four age groups: preschool (3–6 years old) and three follow-up ages (7–9, 10–13, and 14–17 years old). These age groups were chosen to reflect the child’s growing level of independence with respect to food and beverage choices as well as emerging peer influence on those choices. The youngest age group includes children in preschool/kindergarten when parents exert the greatest control over food choices. The second and third age groups include early elementary school and middle school ages, respectively, while the oldest children were those in their high school years who have the greatest level of independence in food choices. For analyses related to the association between 100% fruit juice consumption and subsequent total and whole fruit intakes, preschool fruit juice intake was categorized as < 0.5 cups, 0.5- < 1.0 cups, and ≥ 1.0 cups. To increase power for some analyses, categories of juice intake were collapsed to include < 0.75 cups vs. ≥0.75 cups. Mixed linear regression models for repeated measures data was used to examine the association between juice consumption at 3–6 years of age and total and whole fruit intake as well as HEI scores throughout childhood. Logistic regression modeling was used to estimate the likelihood of meeting dietary guidelines throughout childhood and adolescence. Potential confounding by age, sex, parental education, mother’s BMI, energy intake, physical activity, and television and video viewing time was explored. Only sex was found to confound the results for dietary outcomes and thus was retained in these final models. For the BMI analysis, the final model included age, sex, maternal education, maternal BMI, physical activity, and TV and video viewing time.

## Results

The baseline characteristics of children according to the three categories of preschool fruit juice intake are shown in Table 1. Children who consumed one or more cups of 100% fruit juice per day were slightly younger and had lower energy-adjusted intakes of dietary fat but higher intakes of carbohydrates. In addition, these children consumed more potassium, magnesium, vitamin C, and dietary folate. Finally, education level for the mothers was highest among those consuming the most fruit juice.

**Table 1:** Baseline characteristics of children aged 3–6 years according to preschool consumption of fruit juice

	Categories of 100% fruit juice intake per day						
	< 0.5 cup		0.5- < 1 cup		≥1 cup		
Characteristics	*n = 35*		*n = 35*		*n = 30*		
	Mean	SD	Mean	SD	Mean	SD	*p-trend*
Age (years)	5.2	0.49	5.3	0.59	4.7	0.84	0.0038
BMI (kg/m^2^)	16.3	1.4	16.0	0.92	16.5	1.1	0.1350
Activity (Caltrac counts/hour)	11.3	1.8	10.7	2.0	10.6	1.9	0.3077
Energy (kilocalories)	1519	210	1639	313	1623	240	0.1196
% energy from protein	13.6	1.8	13.7	1.8	13.2	2.0	0.5113
% energy from fat	35.9	3.2	34.4	3.9	31.5	3.8	<.0001
% energy from carbohydrate	52.0	4.1	53.6	4.8	57.0	5.3	0.0002
Calcium (mg/day)	740	212	825	210	748	236	0.2123
Magnesium (mg/day)	180	40	204	43	206	42	0.0232
Potassium (mg/day)	1676	369	1983	412	2150	399	<.0001
Vitamin C (mg/day)	67.7	26.5	89.1	32.0	125.9	49.0	<.0001
Folic acid (mcg/day)	177.8	47.0	194.8	49.6	237.6	64.4	<.0001
Milk (cup-equivalents/day)	1.50	0.61	1.53	0.62	1.42	0.67	0.7629
Added sugar (tsp-equivalents/day)	15.8	4.2	16.3	6.1	14.5	4.4	0.3620
Sugar sweetened beverages, cups/day	0.76	0.48	0.76	0.61	0.60	0.47	0.3695
Whole fruit (cup-equivalents/day)	0.52	0.40	0.65	0.44	0.62	0.34	0.3688
Total Fruit juice (cup-equivalents/day)	0.40	0.20	0.84	0.17	1.63	0.62	<.0001
100% fruit juice (cup-equivalent/day)	0.30	0.16	0.73	0.14	1.51	0.59	<.0001
Healthy Eating Index 2015 Score	48.0	2.0	52.4	6.1	55.0	6.7	0.0002
Number (column percent)							
Gender (% male)	24	(69%)	23	(66%)	14	(47%)	0.0781
Mother’s education (% college)	7	(20%)	14	(40%)	15	(50%)	0.0116

**Fig. 1:**
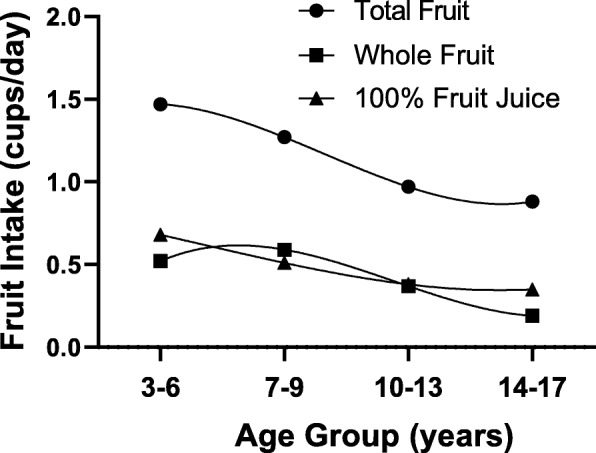
Median intakes of total fruit, whole fruit, and 100% fruit juice throughout childhood (ages 3–17 years) in the Framingham Children’s Study

Figure 1 shows age-specific median intakes of total fruit, whole fruit and fruit juice. During preschool, average total fruit intake was slightly less than 1.5 cups/day; this amount steadily declined to less than a cup per day by mid-adolescence. The median intakes of whole fruit and 100% fruit juice both declined slightly with age.

**Fig. 2:**
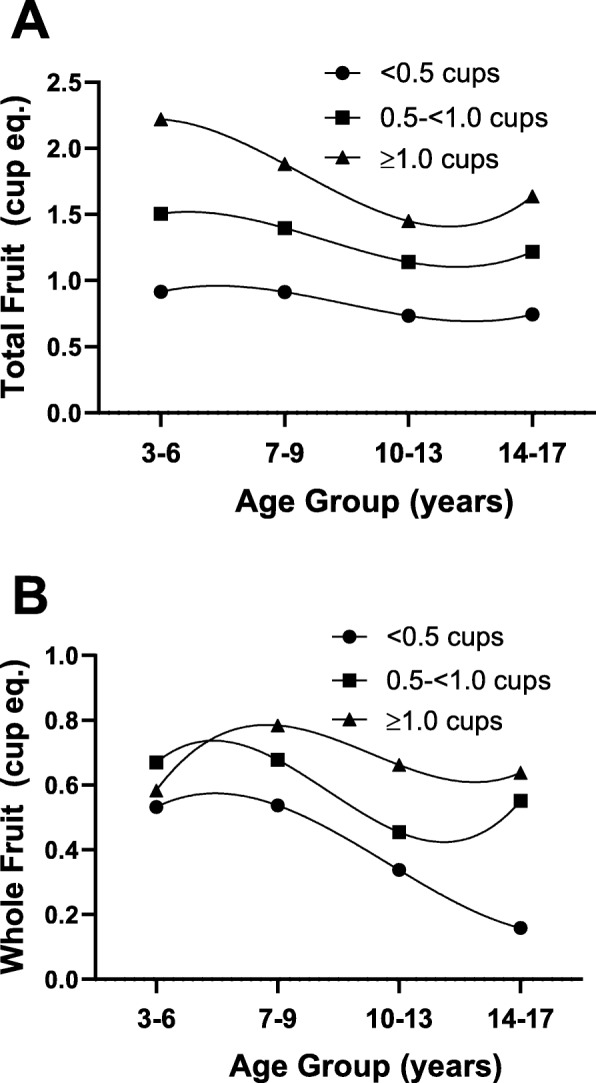
Total (Panel **a**) and whole (Panel **b**) fruit consumption throughout childhood (ages 3–17 years) according to preschool (ages 3–6 years) fruit juice consumption. Results are adjusted for sex

In Fig. 2, children were categorized according to consumption level of 100% fruit juice during preschool years: < 0.5, 0.5- < 1.0, and ≥ 1.0 cups/day. At the end of follow-up (14–17 years of age), children who drank ≥1.0 cups of 100% fruit juice per day (vs. < 0.5 cups/day) during preschool consumed 0.9 cups/day more of total fruit (*p* < 0.0001) (Fig. 2a) and 0.5 cups/day more whole fruit (p < 0.0001) **(**Fig. 2b). Preschool children drinking 0.5- < 1.0 cups/day (vs. < 0.5 cups/day) also consumed significantly more total fruit (*p* = 0.0057) (Fig. 2a) and whole fruit per day (*p* = 0.0009) (Fig. 2b) at 14–17 years of age. Preschoolers consuming < 0.5 cups/day of 100% fruit juice had sharply declining whole fruit intakes starting at age seven compared with those children who consumed more fruit juice at baseline.

In Table 2, preschool children who consumed ≥0.75 (vs. < 0.75) cups/day of 100% fruit juice were much more likely to meet DGA recommendations for whole and total fruit intake. At end of follow-up (14–17 years of age), children who drank ≥0.75 cups/day of fruit juice during preschool (vs. less) were 3.8 times as likely to meet current recommended intakes for whole fruit and total fruit intake (*p* < 0.05).

**Table 2:** Likelihood of meeting total and whole fruit *Dietary Guidelines* by preschool 100% fruit juice intake

Fruit Juice Intake (ages 3–6)	Ages 7–9		Ages 10–13		Ages 14–17	
	OR*	95% CI	OR	95% CI	OR*	95% CI
*Likelihood of Meeting Total Fruit Guidelines*						
< 0.75 cups/day	1.00	–	1.00	–	1.00	–
≥0.75 cups/day	5.71	(2.36, 13.84)	2.14	(0.80, 5.74)	3.83	(1.37, 10.77)
*Likelihood of Meeting Whole Fruit Guidelines*						
< 0.75 cups/day	1.00	–	1.00	–	1.00	–
≥0.75 cups/day	1.95	(0.83, 4.58)	1.92	(0.68, 5.41)	3.80	(1.07, 13.46)
*Adjusted for sex.						

**Fig. 3:**
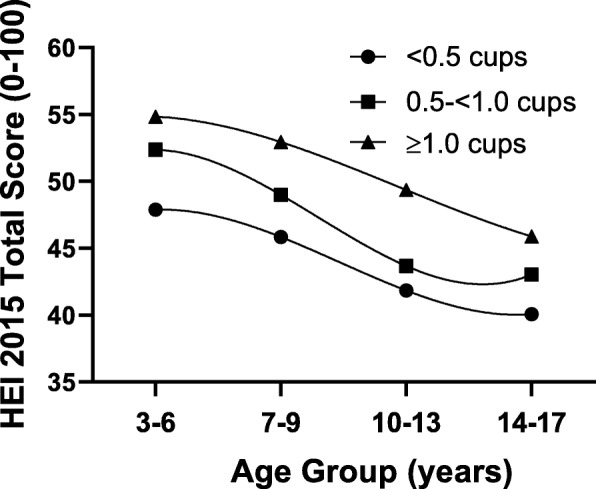
Total Healthy Eating Index-2015 (HEI-2015) scores throughout childhood (ages 3–17 years) according to preschool (ages 3–6 years) fruit juice consumption. Results are adjusted for sex

Figure 3 shows the relation between preschool fruit juice consumption and diet quality throughout childhood as measured by the HEI-2015 total score. First, it is evident that diet quality as measured by the HEI declines steadily throughout childhood. However, children with higher fruit juice intakes during preschool had the highest HEI scores at all ages. At the end of follow-up (14–17 years old), HEI total scores for those with the highest preschool juice intakes (≥1.0 cups/day) were almost 6 points higher than those with the lowest preschool fruit juice intakes (< 0.5 cups/day) (*p* = 0.0044).

**Fig. 4:**
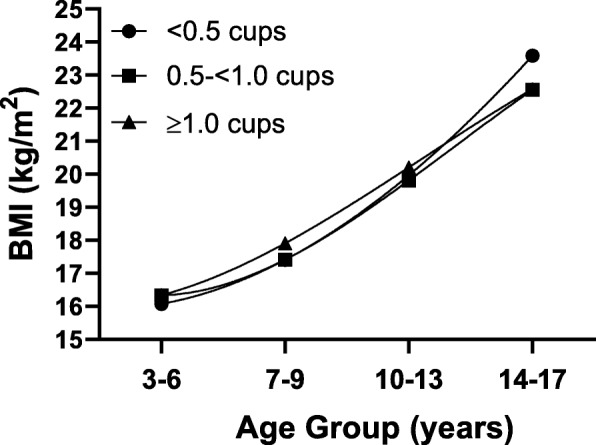
BMI (kg/m2) throughout childhood (ages 3–17 years) according to preschool (ages 3–6 years) fruit juice consumption. Results are adjusted for age, sex, maternal education, baseline BMI, physical activity, and TV and video viewing time

Finally, Fig. 4 shows that 100% fruit juice consumption had no effect on change in BMI throughout childhood after adjusting for age, sex, maternal education, baseline BMI, physical activity, and TV and video viewing time.

## Discussion

The results of the current study provide evidence that higher intake of 100% fruit juice during the preschool years is associated with better diet quality throughout childhood. Findings also confirm that whole fruit consumption declined from early childhood through adolescence in this cohort. These data suggest that preschoolers who consumed more fruit juice in the early years of childhood also consumed more whole fruit at the same time and continue to consume more whole fruit into adolescence.

These data directly address an existing gap in evidence identified by the AAP and others about whether fruit juice consumption promotes healthy eating behaviors and greater intake of whole fruit [8, 24]. This study also suggests that 100% fruit juice consumption during preschool is associated with a higher overall diet quality from preschool into adolescence as measured by the HEI-2015. Additionally, the current results suggest that the nutritional benefits of moderate intakes of fruit juice (above current recommendations) during early childhood are not accompanied by excessive weight gain. Therefore, these results provide no support for the recommendation to eliminate 100% fruit juice from federal child nutrition programs.

These findings are consistent with and extend the results of previous cross-sectional studies of the association between 100% fruit juice consumption and diet quality. Successive analyses of NHANES subjects from early childhood through adolescence have shown positive associations between orange juice [25] and other 100% fruit juices [26, 27] and the intake of key micronutrients and overall diet quality as measured by HEI scores. To the best of our knowledge, the current results are the first to show a longitudinal association of preschool fruit juice consumption and subsequent intake of total and whole fruit as well as higher overall diet quality.

A 2017 meta-analysis of 8 prospective studies that found no clinically significant association between 100% fruit juice and weight gain among children 1–6 years of age and no association at all with weight gain from 7 to 18 years of age [28]. The data in the current study were collected during a time of peak fruit juice consumption among U.S. children [4, 5] as well as the period in which the prevalence of obesity was rising rapidly [29]. Current AAP recommendations for preschool children are to limit intake of 100% fruit juice to no more than 6 oz. per day at 4–6 years of age [8]. The 3–6 year-old children with the highest fruit juice intakes in this study consumed more than 8 oz. per day, allowing us to evaluate potential adverse effects of higher juice consumption on change in BMI in these children. However, no such adverse effect on weight gain was detected.

There is a clear imperative to increase total fruit and whole fruit consumption in younger populations, particularly adolescents. Recent data from Youth Risk Behavior Surveillance System suggests that only 8.5% of high school students meet the current USDA recommendation for fruit intake [30]. Intakes of fruit and vegetables have been found to be associated with beneficial cardiometabolic outcomes in adolescents [31–33]. There is also evidence to suggest that dietary intake patterns are established early in life, track throughout childhood [19], and continue into adulthood [34]. Further, childhood diet appears to be associated with the development and progression of CVD in adulthood [35]. Consequently, the finding that early fruit juice consumption may promote higher intakes of total fruit and whole fruit suggests that moderate juice consumption during the preschool years may have long-term benefits for chronic disease risk.

There are several notable strengths of this longitudinal study. The FCS provides detailed and repeated measures of diet which allow for substantial precision around the estimated effects. Additionally, the intensive level of follow-up from early childhood over 10 years may help to reduce the likelihood of reporting error and/or bias. On the other hand, there are some limitations that must be acknowledged including the small sample size and the homogeneity of the study population. The families in this study are descendants of the original Framingham Heart Study cohort and as such, they are largely of middle-class Caucasian ancestry. There is always the potential for biased reporting of dietary intake in any study. These data, however, were collected over a decade starting in 1987, at a time when there was no negative publicity about juice consumption. The positive association between maternal education and juice intake in the FCS families contrasts with the inverse association between juice consumption and socio-economic status observed a decade later in NHANES [36]. Nonetheless, we found no evidence of confounding of the current results by parental education.

## Conclusions

This longitudinal study adds important evidence addressing the current controversies surrounding the consumption of fruit juice during the preschool years. Early childhood diet plays a crucial role in the establishment of life-long dietary patterns. This study demonstrates that early juice consumption is an important determinant of overall fruit intake and better diet quality in later childhood years without having any adverse effect on energy balance. Thus, this study provides important new evidence that should be considered in the development of future guidelines on fruit juice consumption in early childhood.

## Abbreviations

HEI: Healthy Eating Index
USDA: United States Department of Agriculture
NHANES: National Health and Nutrition Examination Surveys
FCS: Framingham Children’s Study
NDSR: Nutrition Data System for Research
DGA: Dietary Guidelines for Americans
AAP: American Academy of Pediatrics
BMI: Body Mass Index

## References

[1] Slavin JL, Lloyd B (2012). Health benefits of fruits and vegetables. Adv Nutr.

[2] U.S. Department of Agriculture. All About the Fruit Group. Washington, DC. www.choosemyplate.gov/fruit. Published 2018.

[3] U.S. Department of Health and Human Services and U.S. Department of Agriculture. Dietary Guidelines for Americans*, 2015–2020*; 2015. doi:10.1016/S0300-7073(05)71075-6.

[4] Fulgoni VL, Quann EE. National trends in beverage consumption in children from birth to 5 years: analysis of NHANES across three decades. Nutr J. 2012;11:92. 10.1186/1475-2891-11-92. 10.1186/1475-2891-11-92 PMC3551691 23113956

[5] Kim SA, Moore LV, Galuska D (2014). Vital signs: fruit and vegetable intake among children - United States, 2003-2010. MMWR Morb Mortal Wkly Rep.

[6] Banfield EC, Liu Y, Davis JS, Chang S, Frazier-Wood AC (2016). Poor adherence to US dietary guidelines for children and adolescents in the National Health and nutrition examination survey population. J Acad Nutr Diet.

[7] Abrams SA, Daniels SR (2017). Fruit juice and child health. Pediatrics.

[8] Heyman MB, Abrams SA (2017). Fruit juice in infants, children, and adolescents: current recommendations. Pediatrics..

[9] Wojcicki JM, Heyman MB (2012). Reducing childhood obesity by eliminating 100% fruit juice. Am J Public Hlth.

[10] Dennison BA, Rockwell HL, Baker SL (1997). Excess fruit juice consumption by preschool-aged children is associated with short stature and obesity. Pediatrics.

[11] Skinner JD, Carruth BR, Moran J, Houck K, Coletta F (1999). Fruit juice intake is not related to children’s growth. Pediatrics.

[12] Auerbach BJ, Dibey S, Vallila-Buchman P, Kratz M, Krieger J (2018). Review of 100% fruit juice and chronic health conditions: implications for sugar-sweetened beverage policy. Adv Nutr.

[13] Crowe-White K, O’Neil CE, Parrott JS, et al. Impact of 100% fruit juice consumption on diet and weight status of children: an evidence-based review. Crit Rev Food Sci Nutr 2016;56(5):871–884. doi:10.1080/10408398.2015.1061475. 10.1080/10408398.2015.1061475 26091353

[14] O’Neil CE, Nicklas TA. A review of the relationship between 100% fruit juice consumption and weight in children and adolescents. Am J Lifestyle Med 2008;2(4):315–354. doi:10.1177/1559827608317277.

[15] Wang H, Cao G, Prior RL (1996). Total antioxidant capacity of fruits. J Agric Food Chem.

[16] Nicklas TA, O’Neil CE, Kleinman R (2008). Association between 100% juice consumption and nutrient intake and weight of children aged 2 to 11 years. Arch Pediatr Adolesc Med.

[17] Tsao CW, Vasan RS (2015). Cohort profile: the Framingham heart study (FHS): overview of milestones in cardiovascular epidemiology. Int J Epidemiol.

[18] Moore LL, Singer MR, Bradlee ML, et al. Intake of fruits, vegetables, and dairy products in early childhood and subsequent blood pressure change. Epidemiology. 2005;16(1):4–11. doi:10.1097/01.ede.0000147106.32027.3e. 10.1097/01.ede.0000147106.32027.3e 15613939

[19] Singer MR, Moore LL, Garrahie EJ, Ellison RC. The tracking of nutrient intake in young children: the Framingham Children’s study. Am J Public Health 1995;85:1673–1677. doi:10.2105/AJPH.85.12.1673. 10.2105/ajph.85.12.1673 PMC1615722 7503343

[20] Hasnain SR, Singer MR, Bradlee ML, Moore LL. Beverage intake in early childhood and change in body fat from preschool to adolescence. Child Obes 2014;10(1):42–49. http://www.pubmedcentral.nih.gov/articlerender.fcgi?artid=3922282&tool=pmcentrez&rendertype=abstract. 10.1089/chi.2013.0004 PMC3922282 24450382

[21] Schakel SF, Sievert YA, Buzzard IM (1988). Sources of data for developing and maintaining a nutrient database. J Am Diet Assoc.

[22] Tippett KS, Mickle SJ, Goldman JD, et al. *Food and Nutrient Intakes by Individuals in the United States, 1 Day, 1989–1991 Washington, DC,* 1995*. Continuing Survey of Food Intakes by Individuals, 1989–91, Nationwide Food Surveys Rep. No. 91–2*.; 1995. www.ars.usda.gov/ARSUserFiles/80400530/pdf/csfii8991_rep_91-2.pdf.

[23] Reedy J, Lerman JL, Krebs-Smith SM (2018). Evaluation of the healthy eating Index-2015. J Acad Nutr Diet.

[24] Murray RD (2019). 100% fruit juice in child and adolescent dietary patterns. J Am Coll Nutr.

[25] O’Neil CE, Nicklas TA, Rampersaud GC, Fulgoni VL (2011). One hundred percent orange juice consumption is associated with better diet quality, improved nutrient adequacy, and no increased risk for overweight/obesity in children. Nutr Res.

[26] Nicklas TA, O’Neil CE, Fulgoni VL (2015). Consumption of 100% fruit juice is associated with better nutrient intake and diet quality but not with weight status in children: NHANES 2007-2010. Int J Child Heal Nutr.

[27] Maillot M, Rehm CD, Vieux F, Rose CM, Drewnowski A (2018). Beverage consumption patterns among 4-19 y old children in 2009-14 NHANES show that the milk and 100% juice pattern is associated with better diets. Nutr J.

[28] Auerbach BJ, Wolf FM, Hikida A (2017). Fruit juice and change in BMI: a meta-analysis. Pediatrics..

[29] Ogden CL, Carroll MD, Lawman HG (2016). Trends in obesity prevalence among children and adolescents in the United States, 1988-1994 through 2013-2014. JAMA.

[30] Moore LV, Thompson FE, Demissie Z (2017). Percentage of youth meeting federal fruit and vegetable intake recommendations, youth risk behavior surveillance system, United States and 33 states, 2013. J Acad Nutr Diet.

[31] Moore LL, Singer MR, Bradlee ML, Daniels SR (2016). Adolescent dietary intakes predict cardiometabolic risk clustering. Eur J Nutr.

[32] Bradlee ML, Singer MR, Daniels SR, Moore LL (2013). Eating patterns and lipid levels in older adolescent girls. Nutr Metab Cardiovasc Dis.

[33] Mellendick K, Shanahan L, Wideman L, Calkins S, Keane S, Lovelady C (2018). Diets rich in fruits and vegetables are associated with lower cardiovascular disease risk in adolescents. Nutrients.

[34] Mikkilä V, Räsänen L, Raitakari OT, Pietinen P, Viikari J, Willett WC (2005). Consistent dietary patterns identified from childhood to adulthood: the cardiovascular risk in young Finns study. Br J Nutr.

[35] Kaikkonen JE, Mikkilä V, Raitakari OT (2014). Role of childhood food patterns on adult cardiovascular disease risk. Curr Atheroscler Rep.

[36] Drewnowski A, Rehm CD (2015). Socioeconomic gradient in consumption of whole fruit and 100% fruit juice among us children and adults. Nutr J.

